# A systematic review and meta-analysis of voxel-based morphometric studies of fibromyalgia

**DOI:** 10.3389/fnins.2023.1164145

**Published:** 2023-05-09

**Authors:** Ming Xin, Yuzhu Qu, Xingfu Peng, Deliang Zhu, Shirui Cheng

**Affiliations:** ^1^Geriatric Diseases Institute of Chengdu, Department of Rehabilitation, Chengdu Fifth People's Hospital (The Second Clinical Medical College, Affiliated Fifth People's Hospital of Chengdu University of Traditional Chinese Medicine), Chengdu, China; ^2^Acupuncture and Tuina School, Chengdu University of Traditional Chinese Medicine, Chengdu, China; ^3^Acupuncture and Brain Research Center, Chengdu University of Traditional Chinese Medicine, Chengdu, China

**Keywords:** fibromyalgia, neuroimaging, meta-analysis, signed differential mapping, voxel-based morphometry

## Abstract

**Objective:**

Although neuroimaging investigations have revealed significant changes in brain structure in fibromyalgia (FM) patients, these findings are inconsistent. The current study conducted a systematic review and meta-analysis of voxel-based morphometric studies in order to comprehend those alterations in brain structure in FM patients.

**Methods:**

Voxel-based morphometric (VBM) studies published up to January 17, 2023 were searched in the Web of Science, PubMed, EMBASE, Cochrane Library (CENTRAL), China National Knowledge Infrastructure (CNKI), Chongqing VIP, Wanfang Database. Two independent researchers carried out study screening, quality assessment, clinical data and neuroimaging data extraction. The whole-brain voxel-based gray matter (GM) data of FM patients were collected from eligible studies, and meta-analyzed using anisotropic effect size-signed differential mapping (AES-SDM).

**Results:**

Twelve researches were included in this study, including 289 FM patients (mean age: 47.36 years) and 272 HS (mean age: 47.34 years). According to the meta-analysis, FM patients had increased GM in the right postcentral gyrus and left angular gyrus, and decreased GM in the right cingulate gyrus, right paracingulate gyrus, left cerebellum, and left gyrus rectus.

**Conclusion:**

Our study suggests that fibromyalgia patients have altered gray matter in several brain regions that are involved in affective, cognitive functions, and in motor adaptations to pain processing.

## Introduction

Fibromyalgia (FM) is a chronic condition characterized by widespread musculoskeletal pain, along with fatigue, cognitive problems and sleep disturbances (Clauw, 2014; Winslow et al., 2023). FM affects 2 to 4% of the general population on average (Jones et al., 2015), with more female than male being diagnosed (Branco et al., 2010; Winslow et al., 2023). Fibromyalgia patients have high level of health care utilization and high costs associated with medical visits and diagnostic test, which bring heavy economic burden to society and family (Boonen et al., 2005; Pinto et al., 2023). Fibromyalgia is underdiagnosed due to the uncertainty surrounding its etiology (Bair and Krebs, 2020; Gatta et al., 2021).

According to previous studies, FM is a disorder of pain regulation and central sensitization (O'Brien et al., 2018; Siracusa et al., 2021). FM patients showed alterations in gray matter, along with aberrant activity and functional connections in brain regions involving pain processing (Pomares et al., 2017; Aster et al., 2022). Voxel-based morphometric analysis showed that FM patients had increased gray matter in the angular gyrus, cuneus, postcentral gyrus, insula, and putamen (Ceko et al., 2013; Pomares et al., 2017), and decreased gray matter in the bilateral hippocampus, anterior insula, posterior cingulate cortex (PCC), medial prefrontal cortex (MPFC), anterior cingulate cortex (ACC), precentral gyrus, and precuneus (Ceko et al., 2013; Pomares et al., 2017; Boehme et al., 2020). Because of the heterogeneous of the anomalies in gray matters, it is challenging to reconcile the findings of different researches. Although three meta-analyses have been published in 2016, the synthesized results were also heterogeneous (Dehghan et al., 2016; Lin et al., 2016; Shi et al., 2016).

Since 2016, a growing number of neuroimaging researches have helped us better understand the brain underpinnings of FM. Hence, the aim of the present study was to identify the most prominent and replicable GM regions that involved in FM patients from all the whole-brain VBM research published to date using the anisotropic effect size signed differential mapping (AES-SDM), which employs anisotropic kernel during the reconstruction of effect size maps to account for the anisotropy in the spatial covariance of the neuroimaging investigations (Radua et al., 2012a, 2014).

## Methods and analysis

### Search strategy

Systematic searches were conducted from origin to January 17, 2023 in seven electronic databases, including Web of Science, PubMed, EMBASE, Cochrane Library (CENTRAL), China National Knowledge Infrastructure (CNKI), Chongqing VIP, and Wanfang Database. The search terms in PubMed were “fibromyalgia” AND (“voxel-based morphometry” OR “VBM” OR “gray matter” OR “gray matter” OR “voxel wise” OR “voxel-wise”). This search strategy was modified to be suitable for the other six electronic databases. In addition, the review articles and references in the included publications were examined to identify any potential researches that might have been missed in the systematic searches.

### Screening criteria

The article was included if: (1) the abnormalities of gray matter volume or density in adult fibromyalgia patients were investigated using VBM analysis; and (2) the control group were healthy subjects; and (3) the neuroimaging outcomes were reported in three-dimensional coordinates (x, y, z) in Montreal Neurological Institute (MNI) or Talairach space; and (4) magnet strength of the magnetic resonance imaging (MRI) scanner was at least 1.5 Tesla. The article was excluded if: (1) publications were not original article; or (2) the analysis was confined to regions of interests in brain; or (3) the number of participants in any group was fewer than 10.

**Figure 1:**
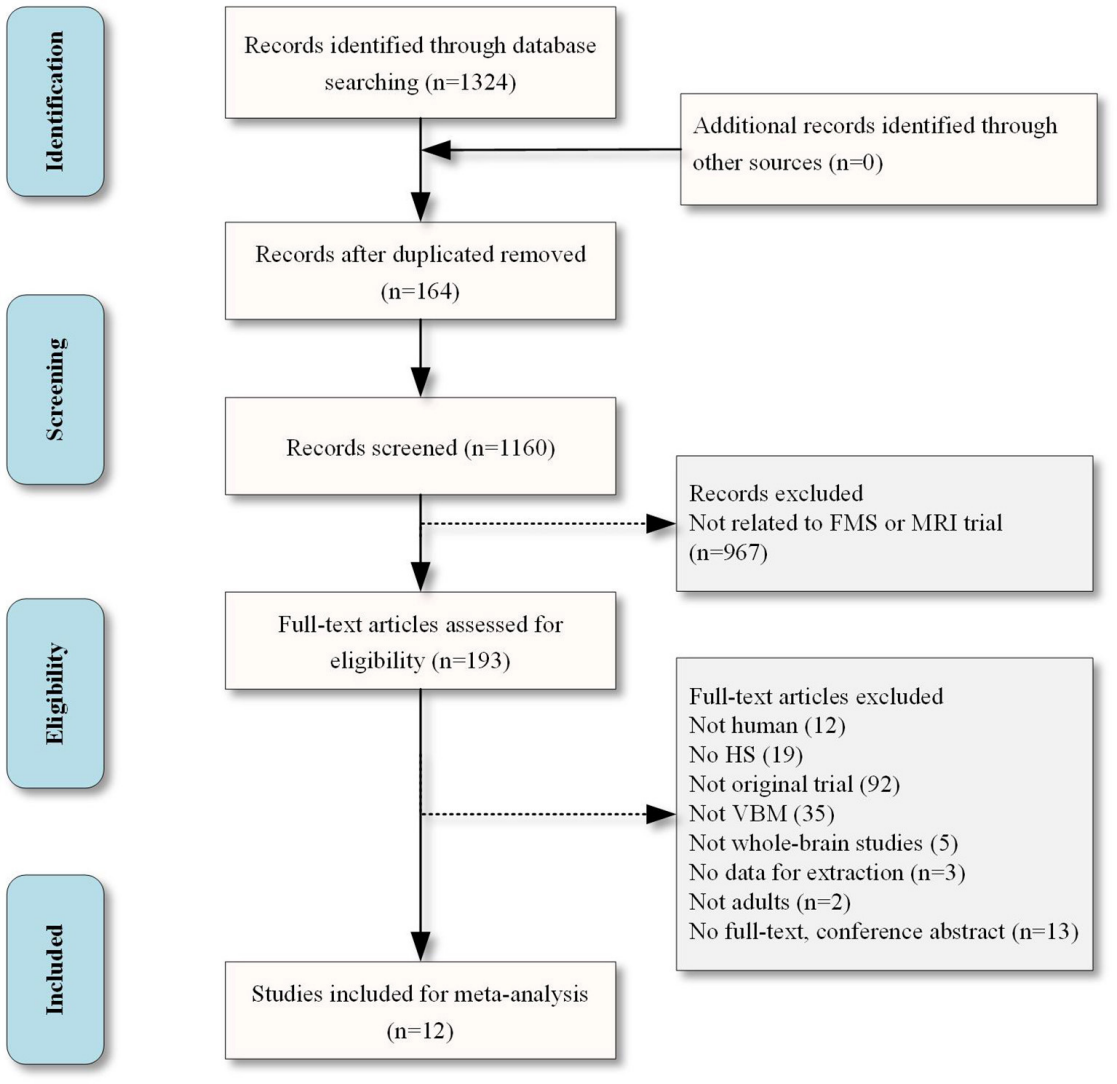
The flow diagram for VBM studies included in the present meta-analysis.

If the data was ambiguous or confusing, the corresponding author of the research was contacted through email. If two or more researches used the same data source, only the article with the largest sample size and most thorough information was included. Only baseline data were included in longitudinal or intervention studies. The current study adhered to the PRISMA (preferred reporting items for systematic review and meta-analysis) guidelines (Figure 1).

### Quality assessment

To evaluate the quality of the included researches, a specialized checklist based on those in prior neuroimaging meta-analyses was used in present study (Supplementary Table S1). The 12-point checklist covered diagnostic procedures, clinical and demographic characteristics, sample size, scanning parameters, analysis methods, and the caliber of the given outcomes. Each research was evaluated separately by two authors (MX, YQ). If there were any rating disputes, the papers were considered by the authors' group to get a decision on a final score.

### Data extraction

The two authors (MX and YQ) independently extracted data from each study, using a predetermined data extraction form. Any discrepancies were discussed in the authors' group in order to be rectified. The authors' name, year of the publication, sample size, age and gender of the study population, disease duration, and the technical information about neuroimaging (MRI scanner, analysis software, full width at half maximum, thresholds, and significant gray matter alterations) were all extracted (Table 1). The peak coordinates in each research were collected following the standards of AES-SDM. In cases of significant results from both corrected and uncorrected thresholds in the VBM statistical analysis of one trial, only the corrected results were collected.

**Table 1:** Demographic and clinical characteristics of subjects in VBM studies included in the meta-analysis.

**Study**	**Number (female)**		**Mean Age (y)**		**Durations (month)**	**MRI scanner**	**Software**	**FWHM**	**Threshold**	**Significant GM alterations**		**Quality score**
	**FM**	**HS**	**FM**	**HS**	**FM**					**Increase**	**Decrease**	
Kuchinad et al. (2007)	10 (10)	10 (10)	52	45	109.2	1.5T	/	10	*p* < 0.05, corrected based on random field theory		**↓**	12
Schmidt-Wilcke et al. (2007)	20 (19)	22 (20)	53.6	50.7	173	1.5T	SPM2	10	*p* < 0.05, corrected	**↑**		11.5
Wood et al. (2009)	30 (30)	20 (20)	42.03	40.05	NA	1.5T	SPM2	12	*p* < 0.0005, uncorrected		**↓**	11
Hsu et al. (2009)	29 (29)	29 (29)	42.6	42.2	153.6	3T	SPM5	10	*p* < 0.05, Bonferroni corrected			12
Ceko et al. (2013)	14 (14)	15 (15)	42.4	43.1	105.6	3T	SPM8, VBM8	8	*p* < 0.05, RFT corrected	**↑**		12
	14 (14)	13 (13)	55.0	55.4	145.2	3T	SPM8, VBM8	8	*p* < 0.05, RFT corrected		**↓**	
Fallon et al. (2013)	16 (16)	15 (15)	38.5	39.4	109.2	3T	SPM8, VBM8	10	*p* < 0.05, FWE corrected	**↑**	**↓**	12
Diaz-Piedra et al. (2016)	23 (23)	23 (23)	41.6	39.7	102.6	3T	SPM8	8	*p* < 0.05, AlphaSim corrected	**↑**	**↓**	12
Pomares et al. (2017)	26 (26)	25 (25)	61	61	NA	3T	SPM8, VBM8	7	*p* < 0.05, corrected based on random field theory	**↑**	**↓**	11
Sundermann et al. (2019)	27 (27)	22 (22)	52.6	52.24	189.12	3T	SPM12, CAT12	8	*p* < 0.05, FWE corrected			12
Boehme et al. (2020)	31 (31)	29 (29)	39.2	42.7	52.8	3T	SPM12, CAT12	NA	*p* < 0.001, uncorrected		**↓**	10
Müller et al. (2021)	32 (32)	32 (32)	50.7	52.5	NA	3T	SPM12	8	*p* < 0.1, FWE corrected			11
Baker et al. (2022)	17 (17)	17 (17)	48.12	48.42	NA	3T	SPM12	8	*p* < 0.01, uncorrected			11

FM, fibromyalgia patients; FWHM, full width at half maximum; HS, healthy subjects; NA, not available; SPM, statistical parametric mapping; T, tesla; VBM, voxel-based morphometry.

### Standard meta-analyses of structural alterations

Alterations in brain structure were subjected to the whole-brain voxel-wise meta-analysis by AES-SDM (www.sdmproject.com/software) (Radua et al., 2012a,b). First, a Gaussian kernel was used to integrate the retrieved peak information to rebuild the effect-size and variance maps, which gave voxels closer to the peaks larger effect sizes. To prevent false-positive results, the assignment's full width at half maximum (FWHM) was fixed at 20 mm (Radua et al., 2012b). Study maps were computed voxel-wise to determining the random-effects mean while taking the sample size, intra-study variability, and between-study heterogeneity into consideration. After determining the meta-analysis means, thresholds were applied using the default parameters (voxel threshold *p* < 0.005, peak height threshold *z* > 1.00, and cluster size threshold > 10 voxels) (Radua et al., 2012b). The meta-analysis effect-size map was then statistically assessed by comparison to a null distribution created using a permutation algorithm. The reproducibility of VBM research results was examined using a leave-one-out Jackknife sensitivity analysis, which did the mean analysis again after methodically removing each research. We furtherly performed a subgroup analysis to rule out any potential heterogeneity originating from different MRI scanning techniques (1.5T or 3.0T scanner). To see if the results could have been influenced by a few or tiny researches, funnel plots of the peaks of the main findings were conducted. Additionally, the Egger test was also conducted to look for any potential publication bias (Radua and Mataix-Cols, 2009).

### Meta-regression analysis

An evaluation of relationships between changes in the brain and subject characteristics (age and duration of FM patients) was carried out using a meta-regression analysis, weighted by sample size and intra- and between-study variances, in order to look for any potential impacts (Radua et al., 2012b). The probability threshold was lowered to 0.005 to reduce the detection of false associations. Results for the slope and one of the regressor's extremes were considered, while results for regions that were not detected in the main analysis were discarded. Fits that were obviously driven by an insufficient number of studies were also discarded by examining the regression plot (Radua et al., 2012b).

## Results

### General information of the included studies

The search strategy resulted in 1,324 articles, and 12 articles were included in this meta-analysis (Figure 1) (Kuchinad et al., 2007; Schmidt-Wilcke et al., 2007; Hsu et al., 2009; Wood et al., 2009; Ceko et al., 2013; Fallon et al., 2013; Diaz-Piedra et al., 2016; Pomares et al., 2017; Sundermann et al., 2019; Boehme et al., 2020; Müller et al., 2021; Baker et al., 2022). One study included two subgroups of fibromyalgia and did separate comparison analyses, so the study was considered separately into two studies for the meta-analysis (Ceko et al., 2013). As a result, the number of studies in meta-analysis was elevated to 13. Among these studies, 9 studies reported gray matter decrease or increase or both in FM patients (Kuchinad et al., 2007; Schmidt-Wilcke et al., 2007; Wood et al., 2009; Ceko et al., 2013; Fallon et al., 2013; Diaz-Piedra et al., 2016; Pomares et al., 2017; Boehme et al., 2020), while 4 studies reported no abnormalities between FM patients and HS (Hsu et al., 2009; Sundermann et al., 2019; Müller et al., 2021; Baker et al., 2022). A total of 561 subjects were considered in this study, including 289 FM patients (mean age: 47.36 years) and 272 HS (mean age: 47.34 years). There was no significant difference in age or gender between the FM patients and HS (*p* > 0.05). The studies had a mean quality score of 11.5 out of a total possible score of 12, indicating that they were of high quality. Details of the literature search and criteria for article inclusion are shown in Figure 1. The clinical variables and technical details of the included studies were presented in Table 1.

### Meta-analyses of GM alterations

**Figure 2:**
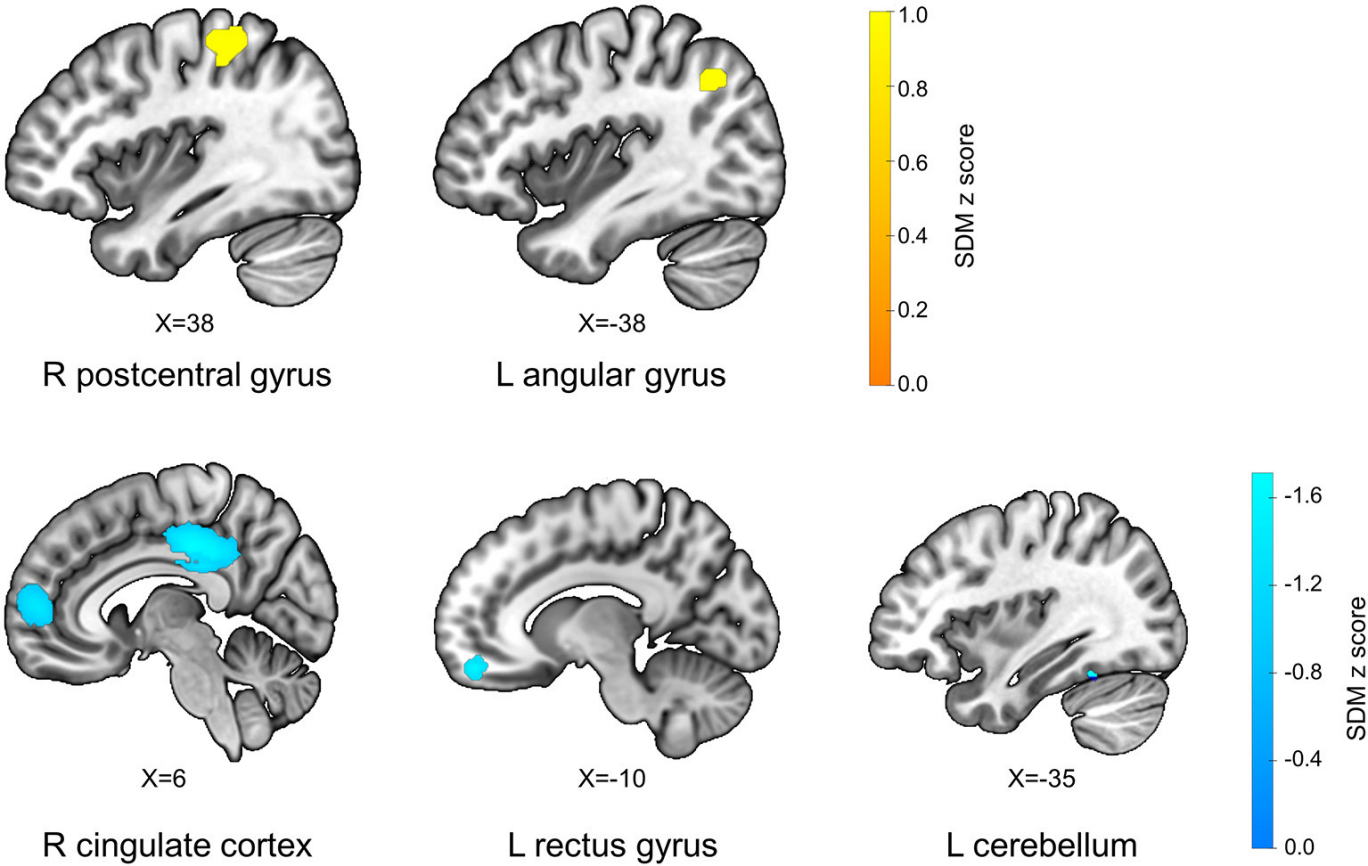
Differences in gray matter between fibromyalgia patients and healthy subjects. L, left; R, right; X, x-axis MNI coordinates of this section of the brain region.

The AES-SDM results showed that FM patients exhibited increased GM in the right postcentral gyrus (*p* = 0.001, *z* = 1.040), and left angular gyrus (*p* = 0.001, *z* = 1.017), and decreased GM in the right cingulate gyrus and right paracingulate gyrus (*p* = 0.000, *z* = −2.105), left cerebellum (*p* = 0.001, *z* = −1.486), and left gyrus rectus (*p* = 0.004, *z* = −1.300) compared with HS (Table 2; Figure 2).

**Table 2:** VBM brain regions showing GM differences between FM patients and HS.

**Regions**	**MNI coordinates**			**SDM *z* score^a^**	***P*-value^b^**	**Number of voxels^c^**	**Cluster breakdown (number of voxels)**	**Heterogeneity**	**Sensitivity**
	**x**	**y**	**z**						
**FM** > **HS**									
R postcentral gyrus	38	−34	56	1.040	0.001	327	R postcentral gyrus, BA2, BA3, BA4, BA40 (256)	No	11/13
							R inferior parietal gyri, BA2, BA40 (30)		
							R precentral gyrus, BA4 (18)		
L angular gyrus	−40	−62	40	1.017	0.001	126	L angular gyrus, BA7, BA39 (57)	No	12/13
**FM**<**HS**									
R cingulate gyrus, R paracingulate gyrus	4	−22	40	−2.105	0.000	1,312	L cingulate / paracingulate gyri, BA23 (434)	No	12/13
							R cingulate / paracingulate gyri, BA23 (545)		
							R median network, cingulum (145)		
							L median network, cingulum (79)		
							L supplementary motor area (14)		
							L posterior cingulate gyrus, BA23 (14)		
							R supplementary motor area (10)		
L cerebellum, hemispheric lobule IV/V	−22	−30	−30	−1.486	0.001	195	L cerebellum, hemispheric lobule IV/V, BA30, BA37 (113)	No	11/13
							Middle cerebellar peduncles (25)		
L gyrus rectus	−2	42	−22	−1.300	0.004	13	L gyrus rectus, BA11 (12)	No	10/13

^*a*^Peak height threshold: z > 1.

^*b*^Voxel probability threshold: p < 0.005.

^*c*^Cluster extent threshold: regions with < 10 voxels are not reported in the cluster breakdown. BA, Brodmann area; FM, fibromyalgia patients; GM, gray matter; HS, healthy subjects; L, left; MNI, Montreal neurological institute; R, right; SDM, signed differential mapping; VBM, voxel-based morphometry.

### Subgroup analysis

The subgroup analysis of VBM studies using 1.5 T scanners revealed structural abnormality in the right cingulate gyrus, right paracingulate gyrus (*p* = 0.001, z = −1.333), left cerebellum, hemispheric lobule IV / V (*p* = 0.001, z = −1.222) of FM patients (Table 3). The subgroup analysis of VBM studies using 3.0 T scanners revealed structural abnormality in the right postcentral gyrus (*p* = 0.000, *z* = 1.162), left angular gyrus (*p* = 0.001, *z* = 1.016), right cingulate gyrus and right paracingulate gyrus (*p* = 0.000, *z* = −1.844), and left gyrus rectus (*p* = 0.002, *z* = −1.452) of FM patients (Table 3).

**Table 3:** Regional differences of FM patients in VBM studies using 1.5 T and 3.0T scanner.

**VBM studies using 1.5 T scanner**							
**Regions**	**MNI coordinates**			**SDM** ***z*** **score**^a^	***P*** **-value** ^b^	**Number of voxels** ^c^	**Cluster breakdown (number of voxels)**
	**x**	**y**	**z**				
**FM**<**HS**							
R cingulate gyrus, R paracingulate gyrus	6	−18	42	−1.333	0.001	92	R median cingulate / paracingulate gyri, BA23 (80)
L cerebellum, hemispheric lobule IV/V	−16	−42	−26	−1.222	0.001	444	L cerebellum, hemispheric lobule IV/V, BA30, BA37 (212)
							L fusiform gyrus, BA37 (71)
							L median network, cingulum (56)
							L parahippocampal gyrus, BA30 (25)
							L lingual gyrus, BA30 (17)
**VBM studies using 3.0 T scanner**							
**FM** > **HS**							
R postcentral gyrus	38	−34	56	1.162	0.000	534	R postcentral gyrus, BA2, BA3, BA4, BA40 (382)
							R precentral gyrus, BA4 (40)
							R inferior parietal gyri, BA2, BA40 (60)
							R superior parietal gyrus, BA2 (11)
L angular gyrus	−36	−64	40	1.016	0.001	200	L angular gyrus, BA7, BA39, BA40 (105)
							L inferior parietal gyri, BA40 (12)
**FM**<**HS**							
R cingulate gyrus, R paracingulate gyrus	4	−22	40	−1.844	0.000	943	R median cingulate / paracingulate gyri, BA23 (333)
							R median network, cingulum (122)
							L cingulate / paracingulate gyri, BA23 (346)
							L median network, cingulum (66)
							Corpus callosum (19)
L gyrus rectus	−2	42	−22	−1.452	0.002	125	Corpus callosum (50)
							L gyrus rectus, BA11 (47)

^*a*^Peak height threshold: z > 1.

^*b*^Voxel probability threshold: p < 0.005.

^*c*^Cluster extent threshold: Regions with < 10 voxels are not reported in the cluster breakdown. BA, Brodmann area; FM, fibromyalgia patients; HS, healthy subjects; L, left; MNI, Montreal neurological institute; R, right; SDM, signed differential mapping; T, tesla; VBM, voxel-based morphometry.

### Meta-regression

A meta-regression analysis was conducted to examine potential confounding variables (mean age, disease duration, and pain intensity). The mean age of FM patients was associated with changed GM in the left angular gyrus (*p* = 0.001, *z* = 2.112), and the right cingulate gyrus, right paracingulate gyrus (*p* = 0.001, *z* = −2.696) in VBM studies (Table 4). The disease duration was associated with altered GM in the left cerebellum (*p* = 0.001, *z* = 2.401) (Table 4). The mean pain intensity of FM patients was associated with abnormal GM in the right cingulate gyrus and right paracingulate gyrus (*p* = 0.000, *z* = 2.054) (Table 4).

**Table 4:** Meta-regression analysis for functional abnormalities in FM patients.

**Regions**	**MNI coordinate**			**SDM *z* score^a^**	***P*-value^b^**	**Number of voxels^c^**
	**x**	**y**	**z**			
**Effect of age**						
L angular gyrus	−40	−62	38	2.112	0.000	84
R cingulate gyrus, R paracingulate gyrus	12	−36	34	−2.696	0.000	236
**Effect of duration**						
L cerebellum, hemispheric lobule IV / V	−22	−30	−34	2.401	0.001	61
**Effect of pain intensity**						
R cingulate gyrus, R paracingulate gyrus	8	−36	32	2.054	0.000	161

^*a*^Peak height threshold: z > 1.

^*b*^Voxel probability threshold: p < 0.005.

^*c*^Cluster extent threshold: Regions with < 10 voxels are not reported in the cluster breakdown. L, left; MNI, Montreal neurological institute; R, right; SDM, signed differential mapping.

### Heterogeneity analysis, sensitivity analysis and publication bias

There was no significant heterogeneity among the VBM studies with GM alterations according to the heterogeneity analysis (*p* > 0.005, Table 2). The leave-one-out Jackknife sensitivity analysis indicated that the left angular gyrus and right cingulate gyrus, right paracingulate gyrus were preserved in 12 combinations (Supplementary Table S2). Publication bias were checked using the funnel plots and the Egger test. The funnel plots demonstrated that the main findings were driven by at least 10 VBM studies (Supplementary Figure S1). Analysis of publication bias revealed that the Egger tests were insignificant in the peaks of the altered brain regions in the VBM meta-analysis (*p* = 0.591).

## Discussion

In order to evaluate the changes of gray matter in FM patients compared with HS, we performed an update meta-analysis using AES-SDM to pool VBM data. FM patients had increased GM in right postcentral gyrus and left angular gyrus, and decreased GM in right cingulate gyrus, right paracingulate gyrus, left cerebellum, and left gyrus rectus. These results remained consistent when each study was eliminated in the Jackknife sensitivity analysis.

Lin et al. included 6 voxel-wise VBM studies (156 FM patients vs. 147 HS), used activation likelihood estimation (ALE) to synthesize the altered gray matter of FM patients, and regional GM loss in left medial prefrontal cortex and right dorsal posterior cingulate cortex in FM patients was discovered (Lin et al., 2016). Shi et al. synthesized the abnormalities of 7 VBM studies (180 FM patients vs. 126 HS), and found GM decreases in the bilateral ACC, MPFC, PCC, paracingulate cortex, and parahippocampal gyrus (Shi et al., 2016). Dehghan et al. synthesized the structural changes in 6 MRI studies, including 4 VBM studies (92 FM patients vs. 92 HS), 1 DTI study and 1 cortical thickness study, and showed variations in the left midcingulate gyrus (Dehghan et al., 2016). In the present study, 12 researches (289 FM patients vs. 272 HS) were analyzed using AES-SDM. Compared with Lin's study, five papers published recently have been added in present study, and the meta-analysis methods were different. The screening criteria used in Shi et al. SDM meta-analysis were inconsistent with our study. Dehghan's study included three kinds of structural MRI researches, and synthesized using ALE. There might be several reasons for the inconsistency of the results in these four meta-analyses. First, only whole-brain gray-matter VBM studies have been included in our study in order to reduce the heterogeneity, and the heterogeneity analysis demonstrated that the main results were robust. Second, the added five studies increased the proportion of 3.0T MRI scanner in present meta-analysis, which influence the results according to our subgroup analysis in studies using 1.5 T scanner and 3.0 T scanner. Third, the SDM and ALE, based on different algorithms, may have a non-negligible impact on the results.

The postcentral gyrus plays a critical role in the perception of pain, its gray matter was increased in people suffering from fibromyalgia (Lutz et al., 2008) and other chronic pain disorders (Ogino et al., 2005). The angular gyrus is involved in visual and sensorimotor information convergence (Prado et al., 2005). The increased gray matter in angular gyrus and postcentral gyrus, implicated in attention to the body and visuo-motor coordination (Macaluso and Maravita, 2010), might relate to increased attentional resources allocated in FM patients to nociceptive and other unpleasant sensory inputs (Schweinhardt et al., 2008). However, none of the three meta-analysis that were published in 2016 showed any increased modification in the gray matter of the postcentral gyrus or the angular gyrus (Dehghan et al., 2016; Lin et al., 2016; Shi et al., 2016), indicating that these alterations may result from the literatures published in recent years (Pomares et al., 2017). The subgroup analysis revealed that the structural MRI scanner (1.5T / 3.0T) had an impact on the increased GM in postcentral gyrus and angular gyrus in FM patients. Three researches using 1.5 T MRI scanners were published in 2007 (Kuchinad et al., 2007; Schmidt-Wilcke et al., 2007) and 2009 (Wood et al., 2009), which had a high proportion of all included researches in the three meta-analysis of VBM studies in 2016 (Dehghan et al., 2016; Lin et al., 2016; Shi et al., 2016). It may also be the reason why there was no such outcome in the three VBM meta-analysis. Additionally, the meat-regression analysis revealed a relationship between age and the increase in gray matter of the angular gyrus in FM patients.

The statistically most robust gray matter declines were observed in the cingulate gyrus and paracingulate gyrus, which were consistent with Dehghan's (Dehghan et al., 2016) and Shi's meta-analysis (Shi et al., 2016). The altered gray matter in the cingulate gyrus was correlated with pain intensity. Cingulate cortex is involved in pain perception, pain modulation, selective attention, error awareness, working memory, and recognition (Kuchinad et al., 2007; Turriziani et al., 2008). Paracingulate gyrus was a significant anatomical marker in the medial prefrontal cortex, and when it is reduced, the ACC around them is increased in gray matter volume (Fornito et al., 2008).

Furthermore, it was noteworthy that gray matter loss in the gyrus rectus was similar to that seen in the cingulate cortex. The anterior cingulate was thought to extend into the frontal lobe through the gyrus rectus (Ballmaier et al., 2004), which may assist to explain why the gray matter abnormalities in both areas are identical. Approximately 30–60% of fibromyalgia patients have psychological comorbidities, which are often characterized by depression and anxiety (Hudson et al., 1985; Boissevain and McCain, 1991; Schmidt-Wilcke and Clauw, 2011). The cingulate cortex and gyrus rectus have also been previously identified as crucial regions implicated in pain catastrophizing and related psychiatric illnesses according to the structural neuroimaging researches conducted to date (Ballmaier et al., 2004; Diaz-Piedra et al., 2016).

In this context, it is also interesting to discuss our findings in the cerebellum. The cerebellum, which is now generally regarded as a cardinal area for pain processing, showed decreased gray matter in FM patients. Researches on both animals and humans has demonstrated that the cerebellum had a role in pain perception and regulation, in addition to motor adaptability, cognitive, and affective activities (Diano et al., 2016; Aster et al., 2022). The activation of cerebellum in FM patients was associated with catastrophizing scores (Gracely et al., 2004), indicating that the cerebellum was involved in pain expectancy and assessment (Schmidt-Wilcke and Clauw, 2011). Several studies have shown increased cerebellar gray matter (Kuchinad et al., 2007; Schmidt-Wilcke et al., 2007), in agreement with the findings from Shi et al. (2016), while Boehme et al. found reduced gray matter density in the cerebellum (Boehme et al., 2020). Studies with larger sample sizes contributed more since the square root of each study's sample size was used to weight the mean map in the SDM analysis. Based on this, the reduction of gray matter in the cerebellum in current meta-analysis was partially influenced by the conclusion of Boehme et al.'s study (Boehme et al., 2020).

## Limitations

There are several limitations that need to be considered when interpreting our results. FM is a chronic pain disorder and shows a wide range of symptoms and severity. The included articles in present study used several pain-related scales that couldn't be converted amongst one another, so the results of meta-regression analysis might not be robust. Besides, only 5 of 13 researches reported the medication used in the FM patients (Ceko et al., 2013; Diaz-Piedra et al., 2016; Boehme et al., 2020; Müller et al., 2021; Baker et al., 2022) and fewer researches reported the emotion state of FM patients, so we can't perform meta-regression analysis to observe the effect of medication taken and emotion state on gray matter changes of FM patients. Furthermore, it is evident that the prevalence of FM in females is obviously higher than that in males (Jones et al., 2015). We are unable to conduct a gender subgroup analysis to compare the differences in gray matter changes between females and males because the majority of the participants in the current study are females.

## Conclusion

In summary, our study suggests that FM patients have altered gray matter in several brain regions that are involved in affective, cognitive functions, and motor adaptations to pain processing. These results might reflect the alterations of chronic pain disorders.

## Data availability statement

The original contributions presented in the study are included in the article/Supplementary material, further inquiries can be directed to the corresponding authors.

## Author contributions

SC and MX contributed to the study conception and design, and conceived the data analysis strategy. MX and YQ acquired the data, collated and analyzed the data, and drafted the manuscript. SC, XP, and DZ discussed, read, and revised the manuscript. All authors approved the publication of this manuscript.
