# Supplementary material for: A systematic review and meta-analysis of voxel-based morphometric studies of fibromyalgia
Source: Front Neurosci. 2023 May 9;17:1164145. doi: 10.3389/fnins.2023.1164145 (PMC10203234; doi:10.3389/fnins.2023.1164145)
Supplement: Supplementary file 1 [file Data_Sheet_1.docx]

# Table S1. Quality Assessment Checklist (1 point per criterion for fully satisfied, 0.5 for partially satisfied, 0 for otherwise)

| **Category 1: Subjects**  **Score** (0/0.5/1) |
| --- |
| 1. Patients were evaluated prospectively, specific diagnostic criteria (American College of Rheumatology criteria for fibromyalgia) were applied, and demographic data were reported. |
| 2. Healthy subjects were evaluated prospectively, and psychiatric and medical illnesses were excluded. |
| 3. Important variables (such as age, gender, illness duration, onset time, medication status, comorbidity, and severity of illness) were reported, either by stratification or statistically. |
| 4. Sample size per group > 10. |
| **Category 2: Methods for image acquisition and analysis** |
| 5. Magnet strength ≥1.5T. |
| 6. MRI slice thickness ≤2 mm. |
| 7. The whole-brain analysis was automatically calculated with no prior regional selection.  8. Coordinates were reported in a standard space. |
| 9. The imaging technique processing was described clearly enough to be reproducible. |
| 10. Measurements were described clearly enough to be reproducible. |
| **Category 3: Results and conclusions** |
| 11. Statistical parameters were provided. |
| 12. Conclusions were consistent with the results obtained and the limitations were discussed. |
| **TOTAL**  /12 |

# Table S2. Sensitivity analysis of VBM meta-analysis

|  | **Increased GM volume Regions** | | |  | **Decreased GM volume Regions** | | |
| --- | --- | --- | --- | --- | --- | --- | --- |
| Studies | R postcentral gyrus | L angular gyrus |  | | R cingulate gyrus, paracingulate gyrus | L cerebellum, hemispheric lobule IV/V | L gyrus rectus |
| Kuchinad et al 2007 | yes | yes |  | | yes | yes | yes |
| Schmidt-Wilcke et al 2007 | yes | yes |  | | yes | yes | yes |
| Wood et al 2009 | yes | yes |  | | yes | no | no |
| Hsu et al 2009 | yes | yes |  | | yes | yes | yes |
| Ceko et al 2013 (1) | yes | yes |  | | yes | yes | yes |
| Ceko et al 2013 (2) | yes | yes |  | | yes | yes | yes |
| Fallon et al 2013 | no | yes |  | | yes | yes | yes |
| Diaz-Piedra et al 2015 | yes | yes |  | | yes | yes | no |
| Pomares et al 2016 | no | no |  | | no | yes | yes |
| Sundermann et al 2019 | yes | yes |  | | yes | yes | yes |
| Boehme et al 2020 | yes | yes |  | | yes | no | no |
| Muller et al 2021 | yes | yes |  | | yes | yes | yes |
| Baker et al 2022 | yes | yes |  | | yes | yes | yes |

Abbreviations: GM, gray matter; L, left; R, right; VBM, voxel-based morphometry.

#
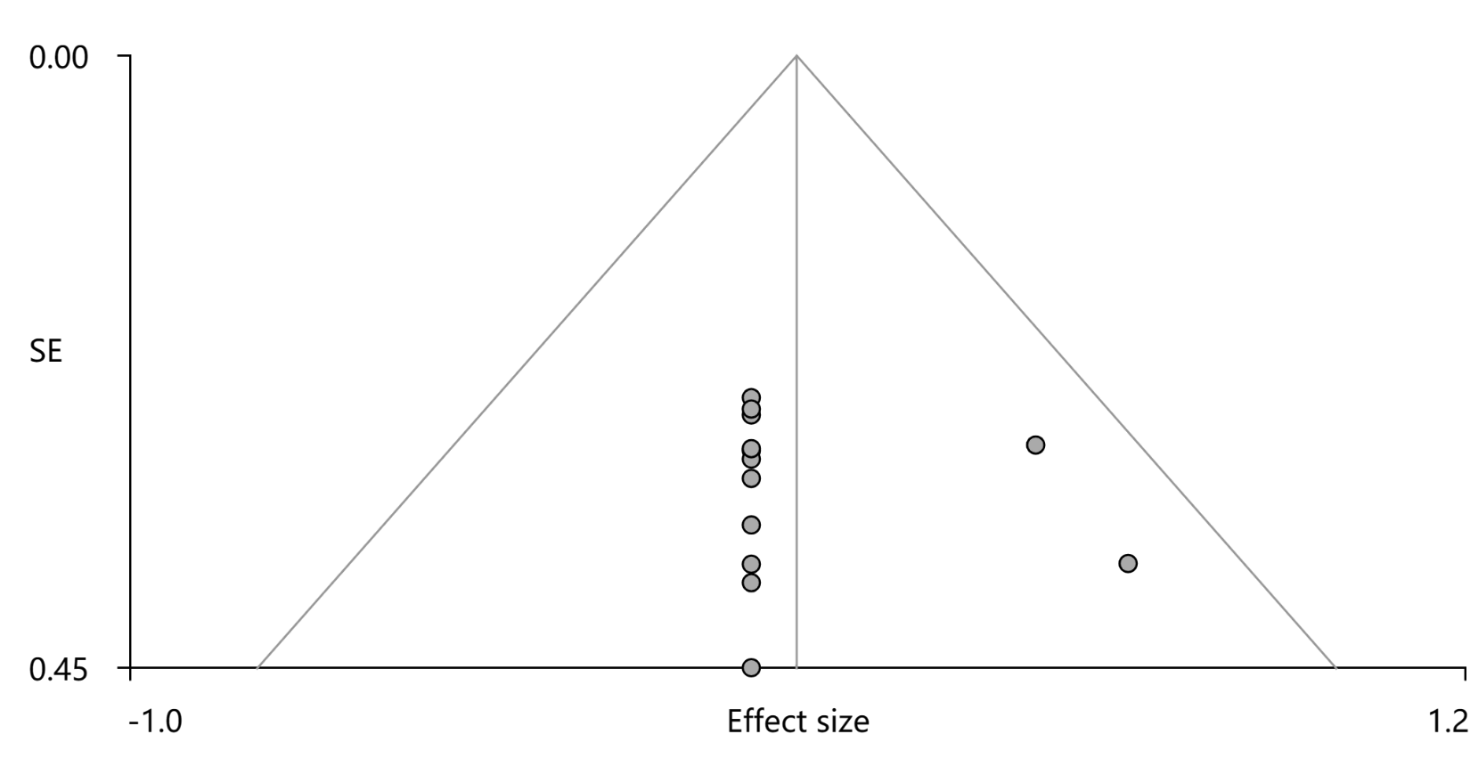
Figure S1. Funnel plots of VBM studies.
